# Low-dose glucocorticoids suppresses ovarian tumor growth and metastasis in an immunocompetent syngeneic mouse model

**DOI:** 10.1371/journal.pone.0178937

**Published:** 2017-06-07

**Authors:** Kai-Ti Lin, Shu-Pin Sun, Jui-I Wu, Lu-Hai Wang

**Affiliations:** 1 Institute of Molecular and Genomic Medicine, National Health Research Institutes, Zhunan, Miaoli County, Taiwan; 2 Department of Life Sciences, National Central University, Jungli District, Taoyuan City, Taiwan; 3 Graduate Institute of Integrated Medicine, China Medical University, Taichung, Taiwan; Southern Illinois University School of Medicine, UNITED STATES

## Abstract

Ovarian cancer has the highest mortality rate among gynecologic malignancies. Despite chemotherapy and surgical debulking options, ovarian cancer recurs and disseminates frequently with a poor prognosis. We previously reported a novel role of glucocorticoids (GCs) in metastatic ovarian cancer by upregulating microRNA-708. In this study, we used an immunocompetent syngeneic mouse model and further evaluated the effect and optimal dosages of GCs in treating metastatic ovarian cancer. The treatment of C57BL/6-derived ovarian cancer ID-8 cells with a synthetic GC, dexamethasone (DEX), induced the expression of microRNA-708, leading to decreased cell migration and invasion through targeting Rap1B. Administration of DEX at a low dose, as low as 5 μg/kg body weight, inhibited the primary tumor size and abdominal metastasis in mice bearing ID-8 cell-derived ovarian tumors. In the treated primary tumors, microRNA-708 was upregulated, whereas some proinflammatory cytokines, namely interleukin (IL)-1β and IL-18, were downregulated. The number of tumor-associated macrophages (TAMs) and myeloid-derived suppressor cells (MDSCs) in the tumor microenvironment were reduced. Overall, our study shows that low-dose GCs can suppress ovarian cancer progression and metastasis likely through not only the upregulation of the metastasis suppressor microRNA-708, but also the modulation of TAMs and MDSCs in the tumor microenvironment.

## Introduction

Ovarian cancer is the fifth most common cause of cancer-related deaths in women [1]. It is categorized into multiple subtypes, with the serous subtype of epithelial-derived ovarian cancer (EOC) being the predominant and most lethal form [2]. The high morbidity of EOC is due to the lack of early detection methods and poor responses to treatment when the tumor spreads. The 5-year survival rate decreases from 90% in the early stage to 14% when the tumors metastasize to distant organs [3]. Many patients with ovarian cancer transiently respond to the frequently used first-line platinum-based chemotherapy; however, most develop recurrent chemoresistant disease within 18 months [4]. Clinically approved drugs targeting metastasis in patients with advanced metastatic ovarian cancer are currently unavailable. Studies devising novel therapeutic strategies against ovarian cancer metastasis are urgently warranted to improve patient outcomes.

Glucocorticoids (GCs) markedly affect the metabolism, differentiation, proliferation, inflammation, and survival of cells [5]. Synthetic GCs, such as dexamethasone (DEX), have been widely used in clinical oncology during chemotherapy and radiotherapy to alleviate side effects in nonhematological cancer types [6]. However, no comprehensive study has investigated the marked complexity of GC-mediated signaling in tumor progression and metastasis [7]. We previously reported that GC-mediated signaling inhibits metastasis in ovarian cancer and specifically identified a novel regulatory mechanism of GCs in suppressing cancer metastasis by regulating a metastasis suppressor microRNA (miRNA), miR-708 [8]. However, in the mouse model used in our previous study, we orthotopically injected human ovarian cancer cells into severely compromised immunodeficient (SCID) mice, which may not reveal the possible effects of GCs in the immune modulation of the host, particularly in the tumor microenvironment; which is likely to play an important role in the progression and metastasis of ovarian cancer.

Progress in ovarian cancer research has been hampered by a lack of genetically manipulated mouse models of spontaneous ovarian tumors. However, a mouse model of spontaneous *ex vivo* transformation of EOC was established [9] and has been extensively used in preclinical studies of therapeutic agents [10–12] and the tumor microenvironment [13, 14]. Taking advantage of this well-established mouse model, here we further evaluated the role of GCs in ovarian cancer progression in mice with an intact immune system.

We found that treatment with DEX induced the expression of miR-708, leading to the suppression of Rap1B, which resulted in the reduction of cell migration and invasion in a mouse-derived EOC cell line, ID-8 cells. Treatments with low-dose DEX significantly suppressed the primary ovarian tumor size and abdominal metastasis in mice bearing ID-8 cell-derived ovarian tumors. MiR-708 expression was upregulated in the DEX-treated mice, whereas some proinflammatory cytokines as well as the number of tumor-associated macrophages (TAMs) and myeloid-derived suppressor cells (MDSCs) in the tumor microenvironment were reduced in these mice. Overall, these findings support and extend our previous results, indicating that low-dose GCs suppress ovarian cancer progression and metastasis in an immunocompetent syngeneic mouse model, and this suppression may not only be due to the induction of miR-708, but also the inhibition of TAMs and MDSCs at the tumor microenvironment.

## Material and methods

### Cell culture and transfection

ID-8 cells (a mouse EOC cell line established in 2006 and recently obtained from Dr. Kathy Roby, Kansas University Medical Center) were maintained in Dulbecco modified Eagle medium (DMEM; Invitrogen), containing 4% fetal bovine serum (FBS; Biological Industries, Israel) and 1× penicillin–streptomycin, at 37°C in 5% CO_2_. The cells were transfected using Lipofectamine 2000 (Invitrogen), and 20 nM was used for each miRNA, anti-miRNA, or small interfering RNA (siRNA) transfection. ID-8 cells stably expressing green fluorescent protein (GFP)–luciferase were generated by infection with lentiviral particles encoding GFP–luciferase genes and were subsequently selected from mass culture resistant to puromycin (Sigma-Aldrich) treatment.

### Nucleotides and reagents

The precursor miR-708 was purchased from Applied Biosystems (Carlsbad, CA), and anti-miR-708 was obtained from GeneDireX (Vegas, NV). The Rap1B-specific siRNA was purchased from MDBio Inc (QingDao, China). Detailed sequences are listed in S1 Table. DEX was obtained from Sigma–Aldrich.

### Quantitative real-time polymerase chain reaction for genes and miRNAs

RNA was extracted from cells using TRIzol (Invitrogen), according to the manufacturer’s protocols. First-strand cDNA was generated using ReverTra Ace (Toyobo, Japan) by using oligo-dT or corresponding miRNA reverse transcription (RT) primers. Real-time RT-polymerase chain reaction (PCR) was performed using a CFX96 real-time PCR detection system (BioRad). The KAPA SYBR FAST Universal qPCR Kit (KAPA Biosystems, MA) was used for gene detection. This kit in addition to Universal Probe Library #21 (Roche Applied Science) was used for miRNA detection. The mRNA levels were normalized to those of actin, whereas miRNA levels were normalized to those of U6. All primer sequences used in this study are shown in S1 Table. The experiments were performed in triplicate. For cell culture experiment, data are normalized mean ± SD from three independent experiments. The level of DMSO control group was normalized to one. ANOVA followed by Dunnett's post-hoc test was used for the statistical test (***P < 0.001). For xenograft tumor analysis, data are normalized mean ± SEM from individual tumors (3–6 per group). The level of control group was normalized to one. ANOVA followed by Dunnett's post-hoc test was used for the statistical test when multiple means were compared (*P<0.05; **P<0.01).

### Western blot analysis

The cells were lysed in 10 mM Tris buffer, pH 7.4, containing 0.15 M NaCl, 1% Triton X-100, 1 mM ethylenediaminetetra-acetic acid, and a protease inhibitor mixture (Roche). The cell lysates were resolved in an 8%–12% sodium dodecyl sulfate–polyacrylamide gel, transferred onto polyvinylidene fluoride membranes, and probed with antibodies. Antibodies against Rap1B were obtained from Cell Signaling Technology; anti-actin was purchased from Santa Cruz Biotechnology.

### Transwell cell migration and cell invasion assays

Cell migration and invasion Boyden chamber assays were performed as previously described [15]. Cell migration was assayed in 8.0-mm Falcon cell culture inserts (Corning), and for the cell invasion assay, the BD Biocoat Matrigel Invasion Chamber was used (Corning). Briefly, 5 x 10^4^ cells were suspended in DMEM (300 μL) and placed in the upper transwell in an area of 0.3 cm^2^. The bottom well was filled with 500 μL of DMEM supplemented with 10% FBS. After incubation for 6 h (migration) and 16 h (invasion), the cells on the upper side of the inserts were removed using cotton swabs and those on the bottom were fixed and stained with crystal violet. Photos of three regions were captured, and the numbers of cells were counted using Image J (National Institutes of Health, USA). All experiments were performed in triplicate. Data are normalized mean ± SD from three independent experiments. The level of DMSO control group was normalized to one. ANOVA followed by Dunnett's post-hoc test (for 3 or more groups) or student t test (for 2 groups) was used for the statistical test (*P<0.05; **P<0.01; ***P < 0.001).

### Cell proliferation assay

Cell proliferation assay was measured with the CellTiter 96 Aqueous One Solution cell proliferation assay (MTS assay; Promega). Assay was performed according to the methods described in the manufacturer’s manual. Briefly, ID-8 cells (1x10^3^ cells) were seeded in 96-well plates and incubated for various times, at defined time points, 20 μL of the CellTiter 96 AQueous One Solution Reagent was added and incubated for 2 hrs at 37℃. The quantity of formazan product, which is directly proportional to the number of living cells in the culture, was measured by absorbance at 490 nm with 96-well plate reader. Data are normalized mean ± SD combined from three independent experiments. Data presented as fold change from untreated (day 0) expression level.

### Mouse orthotopic metastasis model

In orthotopic implantation, GFP–luciferase-labeled ID-8 cells (1 x 10^6^) were harvested and resuspended in 20 μL of phosphate-buffered saline (PBS) containing 50% Matrigel (BD Biosciences). The cells were intrabursally injected into the ovary of 6- to 8-week-old C57BL/6 female mice obtained from BioLASCO (Taiwan). The animal facility personnel from National Health and Research Institutes monitored the animals daily, checking for levels of food and water in each cage. Mice were also physically checked three times a week by the researcher. The basic animal maintenance includes housing the mice in cages (four per cage), sufficient diet, water and bedding. The cages were cleaned and sanitized on regular basis. In the DEX treatment experiments, 5 mg/mL DEX was dissolved in ethanol and further diluted in PBS for an intraperitoneal injection of 100, 50, or 5 μg/kg three times a week starting from day 5 after cell implantation. A protocol was in place for humane endpoint if animals became severely ill prior to experimental endpoint, the mice will be euthanized. Humane endpoints were defined as follows: 20% weight loss, cough hair coat, jaundice and/or anemia, coughing, labored breathing, nasal discharge, neurological signs (frequent seizure activity, paralysis, ataxia), prolapse, self-induced trauma, any condition interfering with eating or drinking, excessive or prolonged hyperthermia or hypothermia, tumor size over 20mm in diameter or tumor weight over 4000mg. However, none of the animals met the criteria for euthanasia before the experimental endpoint. We also had a protocol in place to alleviate animal suffering, including use of anesthetics and analgesics. Tumor growth and abdominal metastases were monitored through live animal bioluminescence imaging (BLI) after day 90 of the implantation (Caliper IVIS system, PerkinElmer). The mice were intraperitoneally injected with luciferin, and 10 min later, they were euthanized by CO_2_ inhalation and individual abdominal organ metastases were quantified using *ex vivo* BLI signals of each organ. All procedures of our mouse study were performed according to approved protocol by Institutional Animal Care and Use Committee of National Health and Research Institutes (NHRI-IACUC-103048). The *ex-vivo* BLI measurements were used to quantitatively evaluate individual tumor growth and abdominal organ metastases. Data are presented as the mean ± SEM (n = 7–14 mice per group; data were combined from two experiments). ANOVA followed by Dunnett's post-hoc test was used for the statistical analysis when more than two means were compared (*P < 0.05; **P < 0.01).

### Immunohistochemistry

Paraffin-embedded tissue sections of mouse ID-8 ovarian tumor specimens were obtained from mouse experiments. One slide with maximum tumor area per mouse was sent to pathology core laboratory (National Health Research Institutes, Taiwan) for further staining with F4/80 (Abcam) or GR-1 (BioLegend) antibody by using the automatic slide stainer BenchMark XT (Ventana Medical Systems, AZ). All immunostained slides were digitalized with Panoramic Scan (3DHISTECH Ltd., Budapest, Hungary). Each slide is from one individual tumor, and positively stained cells among the tumor areas in one single slide were counted and divided by the tumor area (mm^2^). Four or five mice, i.e. 4 to 5 slides, were analyzed per group. Student t test was used for the statistical test (*P<0.05).

## Results

### MicroRNA-708 expression inhibits mouse ovarian cancer cell Rap1B expression, migration and invasion

**Fig 1:**
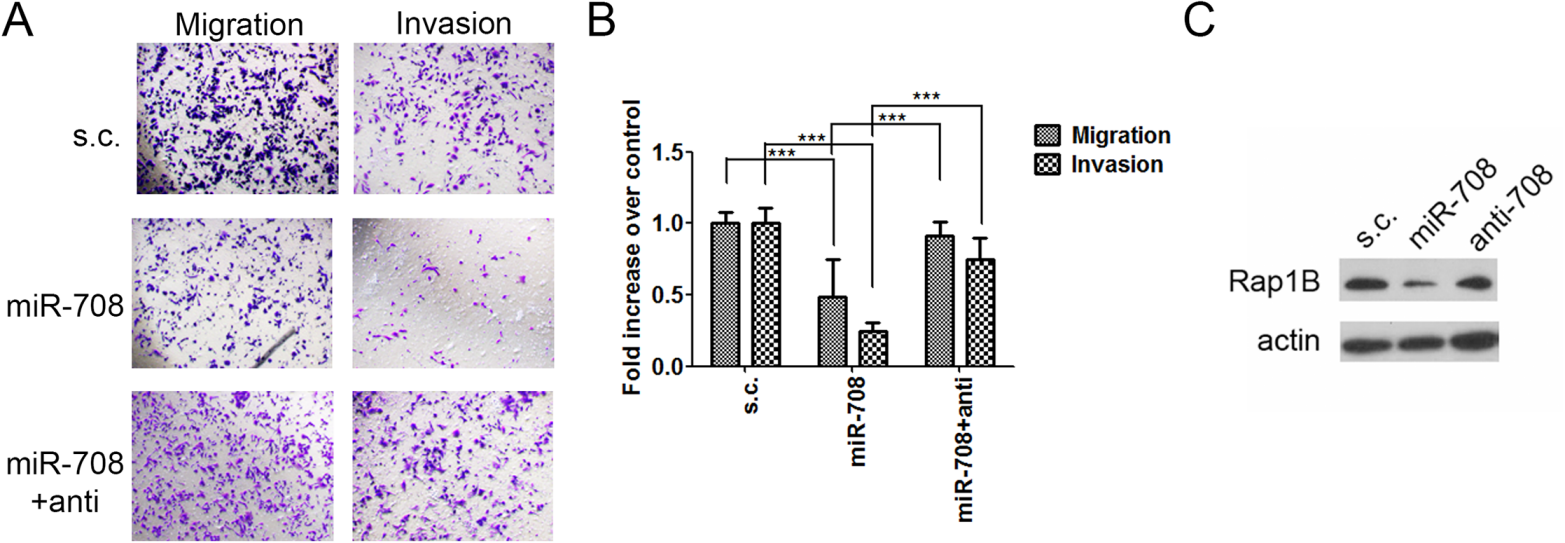
MicroRNA-708 suppresses cell migration and invasion as well as Rap1B expression in ID-8 cells. (A and B) ID-8 cells transfected with miR-708, miR-708 plus anti-miR-708, or a scramble control (s.c.) were incubated for migration (6 h) and invasion (16 h) assays. (A) Representative images of the bottom surface are shown. (B) The cells in three random microscopic fields (200×) were enumerated for each group. Data are normalized mean ± SD from three independent experiments. The percentage of migration/invasion obtained for control group was normalized to one. ANOVA followed by Dunnett's post-hoc test was used for the statistical test (***P < 0.001). (C) Rap1B expression in ID-8 cells transfected with miR-708, miR-708 plus anti-miR-708, or s.c.

We previously demonstrated that DEX can induce miR-708, which is downregulated in the advanced stages of ovarian cancer, to inhibit the migration and invasion of human ovarian cancer cells in culture as well as the growth and metastasis of tumors derived from them in an immunocompromised xenograft mouse model [8]. Because GCs, such as DEX, can modulate the immune system, we wished to employ an immune competent syngeneic mouse model to assess the miR-708 induction and tumor inhibitory effects of DEX. The mouse ovary epithelial ID-8 cancer line was derived from C57BL/6 mice and has been used in syngeneic mouse model for ovarian cancer [9]. We first examined whether miR-708 can suppress ID-8 cell migration and invasion, which were assessed using Boyden chambers transwell assays, as previously described [8]. MiR-708 expression in ID-8 cells significantly inhibited their migration and invasion abilities (Fig 1A and 1B), supporting our hypothesis that miR-708 plays a suppressive role in the migration and invasion of mouse-derived epithelial ovary cancer cells.

MicroRNA-708 has been reported to suppress cell progression and metastasis in certain cancer types [8, 16–18] by inhibiting different targets, namely Rap1B [8], neuronatin [18], or survivin [16]. Rap1B, a member of the Ras oncogene family, regulates cell adhesion and motility through integrin-mediated signaling [19]. We previously reported that miR-708 suppressed cell spreading and adhesion by regulating focal adhesion formation mediated by Rap1B. Restoring Rap1B expression in human EOC cells abrogated miR-708-mediated suppression of migration and invasion in cell culture and abdominal metastasis in an orthotopic mouse model [8]. Therefore, we next determined whether miR-708 regulates Rap1B expression in ID-8 cells. The results revealed that miR-708 expression in ID-8 cells reduced Rap1B (Fig 1C), and this inhibition was abrogated by anti-miR-708 coexpression.

### MicroRNA-708 suppresses ovarian cancer cell migration and invasion via downregulation of Rap1B

**Fig 2:**
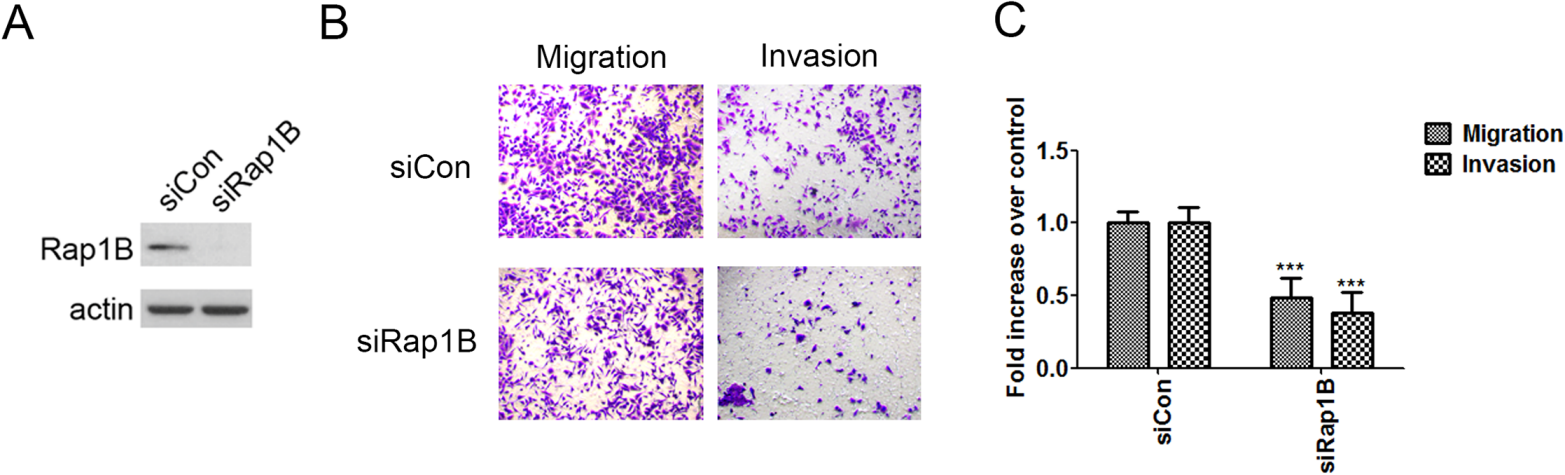
Rap1B knockdown in ID-8 cells inhibits their migration and invasion. (A) Rap1B expression in ID-8 cells upon Rap1B or control siRNA transfection. (B and C) ID-8 cells transfected with Rap1B or control siRNA were incubated for migration (6 h) and invasion (16 h) assays. (B) Representative images of the bottom surface are shown. (C) The cells in three random microscopic fields (200×) were enumerated for each group. Data are normalized mean ± SD from three independent experiments. The percentage of migration/invasion obtained for control group was normalized to one. Student *t* test was used for the statistical analysis (***P < 0.001).

To assess whether Rap1B serves as a downstream effector of the miR-708-mediated inhibition of ID-8 cell motility and invasiveness, we determined the role of Rap1B in cell migration and invasion. The depletion of Rap1B by siRNA (Fig 2A) reduced both the migration and invasion abilities of ID-8 cells (Fig 2B and 2C), suggesting the key role of Rap1B in the miR-708-mediated suppression of cell migration and invasion.

### Glucocorticoid suppresses cell migration and invasion by upregulating miRNA-708 transcription in ID-8 cells

**Fig 3:**
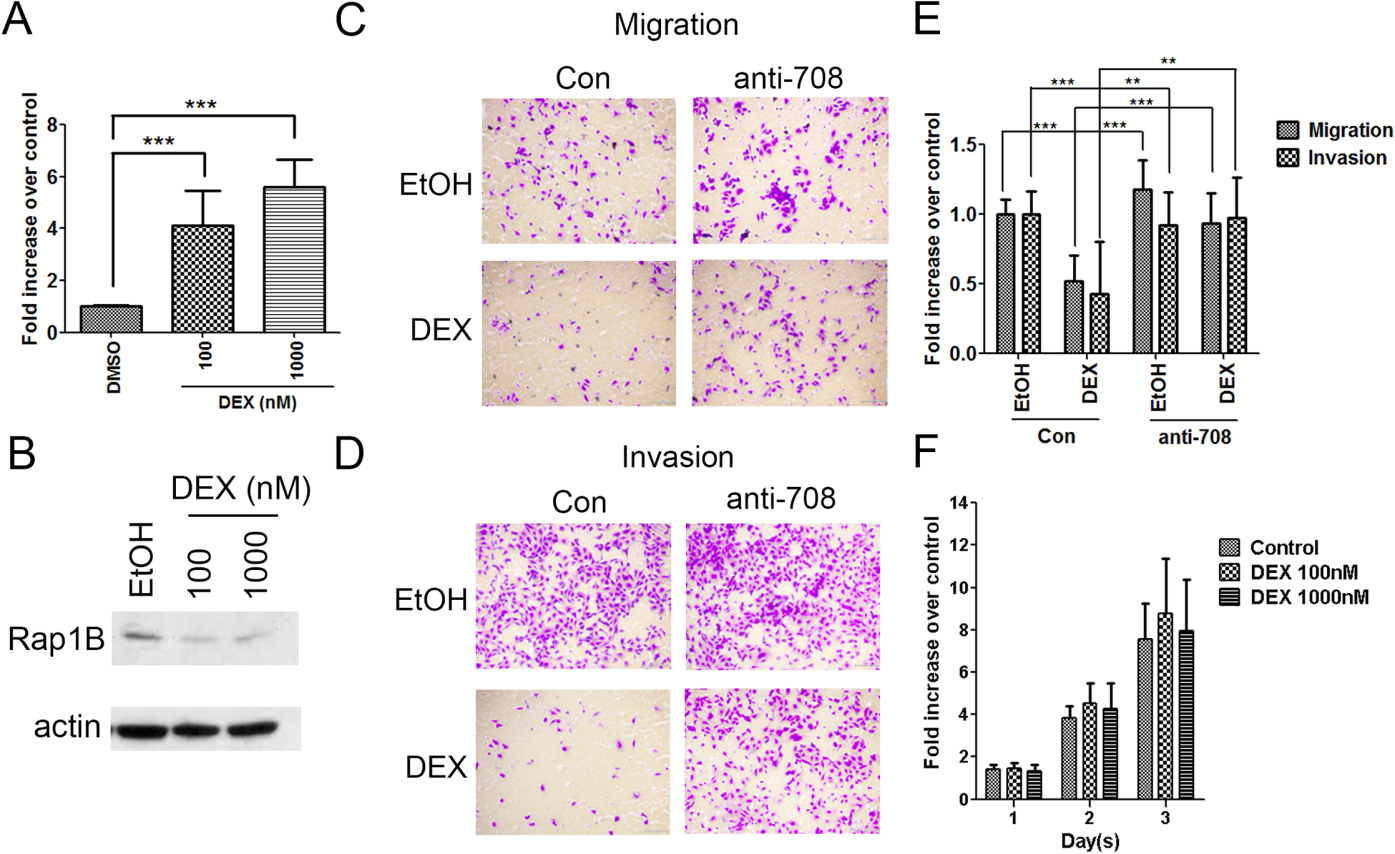
GC signaling reduces the migration and invasion abilities of ID-8 cells through miR-708 induction. (A) miR-708 expression in ID-8 cells after 72 h of treatment with an increasing amount of DEX (100–1000nM). Data are normalized mean ± SD from three independent experiments. The level of DMSO control group was normalized to one. ANOVA followed by Dunnett's post-hoc test was used for the statistical test (***P < 0.001). (B) Western blot analysis showing Rap1B expression in ID-8 cells upon treatment with 100–1000nM DEX for 48 h. β-actin was used as the loading control. (C–E) ID-8 cells transfected with anti-miR-708 or a control were treated with 1000nM DEX overnight and subsequently subjected to migration (6 h) and invasion (16 h) assays. Representative images of the migration (C) and invasion (D) assays are shown. (E) The cells in three random microscopic fields (200×) were enumerated for each group. Data are normalized mean ± SD combined from three independent experiments. The percentage of migration/invasion obtained for control group was normalized to one. ANOVA followed by Dunnett's post-hoc test was used for the statistical test (**P < 0.01; ***P < 0.001). (F) Cell proliferation assay was performed in ID-8 cells by MTS reagent. 1x10^3^ cells were cultured and assayed for proliferation in 96 wells. In the next day, ID-8 cells were treated with 100–1000nM DEX for 3 days. Data are normalized mean ± SD combined from three independent experiments. Data presented as fold change from untreated (day 0) expression level.

We previously reported that GCs induced miR-708 transcription in human ovarian cancer cells [8]. Consistent with that observation, we observed that DEX treatment significantly induced miR-708 expression in ID-8 cells (Fig 3A). Concordantly, DEX treatment reduced Rap1B expression (Fig 3B). We further assessed whether migration and invasion abilities were mediated by GC signaling through miR-708 expression. DEX significantly reduced both abilities of the ID-8 cells, and the depletion of the endogenous miR-708 by anti-miR-708 abrogated this DEX-mediated inhibition of cell migration and invasion (Fig 3C–3E). On the other hand, cell proliferation rates upon DEX treatment were not affected (Fig 3F). Overall, our results revealed that GCs suppress cell migration and invasion, mainly via the miR-708–Rap1B signaling pathways in ID-8 cells.

### Low-dose dexamethasone treatment suppresses ovarian cancer abdominal metastasis in an immunocompetent syngeneic mouse model

**Fig 4:**
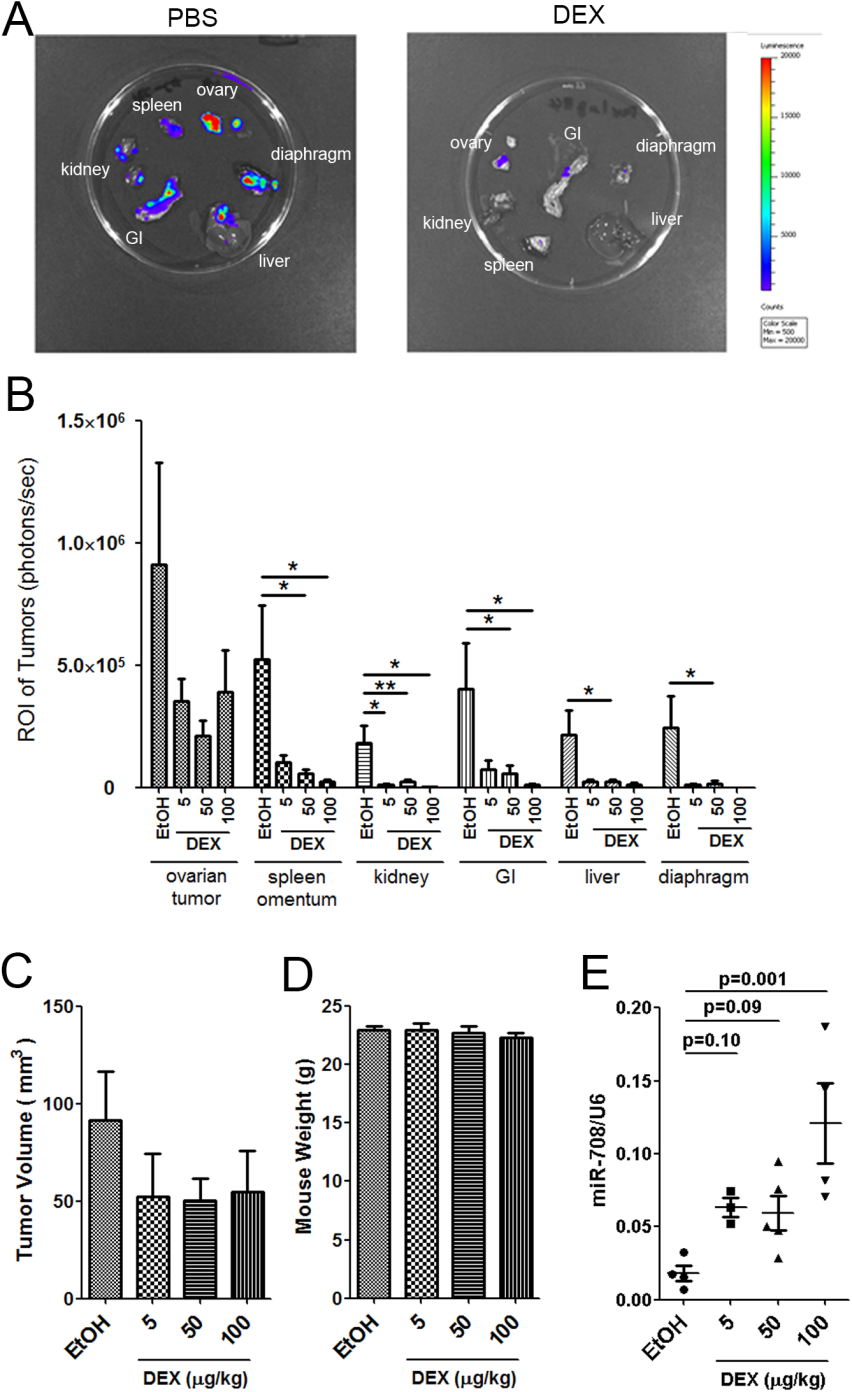
Low-dose DEX suppresses ovarian tumor growth and abdominal metastasis. (A) ID-8 cells stably expressing pSuper-GFP–Luc were orthotopically injected into mouse ovary bursa. Five days after implantation, we initiated intraperitoneal DEX administration (5, 50, or 100 μg/kg) three times a week until day 90. The kinetics of individual abdominal organ metastasis was monitored through *ex vivo* BLI. Representative bioluminescence images at day 90 after implantation are shown. (B) Quantification results of individual abdominal organ metastases by BLI measurements. Data are presented as the mean ± SEM (n = 7–14 mice per group; data were combined from two experiments). ANOVA followed by Dunnett's post-hoc test was used for the statistical test (*P < 0.05; **P < 0.01). (C, D) The tumor volume (C) and the mouse weight (D) were compared. Data are presented as mean ± SEM (n = 7–14 mice per group; data were combined from two experiments). (E) Real-time RT-PCR showed miR-708 expression in primary orthotopically implanted ovarian tumors with a control or DEX treatment. Data represent as mean ± SEM (n = 3–6 mice per group). ANOVA followed by Dunnett's post-hoc test was used for the statistical test.

A study reported that the orthotopic grafting of ID-8 cells into syngeneic mouse ovarian bursa induced the formation of epithelial ovarian tumors as well as secondary lesions throughout the peritoneal cavity and extensive abdominal ascites [13]. Those characteristics of the model closely resemble the pathology known to be associated with human ovarian cancer. Moreover, this syngeneic mouse model is suitable for studying the tumor microenvironment with regard to the effect of DEX on immune components [13, 14]. Therefore, we used this syngeneic mouse model and determined the effects of GCs in ovarian cancer progression, abdominal metastasis, and the tumor microenvironment. GFP–luciferase labeled ID-8 cells were orthotopically implanted into the ovarian bursa of the C57BL/6 mice. Five days after implantation, we initiated intraperitoneal DEX administration (100, 50, or 5 μg/kg) three times a week until day 90. All mice survived at the end of experiment except two deaths. The human equivalent dose of DEX was 8, 4, or 0.4 μg/kg each time [20], which is approximately 30–500-fold less than the common clinical dosages (8–20 mg per administration) used in patients with cancer. *Ex vivo* BLI was conducted with multiple abdominal tissues excised from each mouse after the final *in vivo* imaging session. These tissues included the ovary tumor, omentum, spleen, liver, kidney, gastrointestinal (GI) tract, and diaphragm. Representative *ex vivo* bioluminescence images of a primary ovary tumor and abdominal metastasis are shown in Fig 4A. After 3 months of DEX treatment, the DEX-treated group showed a smaller tumor volume than did the control group, even with the lowest concentration (5 μg/kg), when each single dose of DEX treatment group was compared with the control. However, due to individual variation and the relatively small number of mice per group, the decrease of tumor size was not significantly proportional to the DEX doses when all treatment groups were compared together with the control (Fig 4B and 4C). This could also mean that 5 μg/kg had already reached the maximal effect on tumor growth inhibition. The mice weight were not affected by all doses of DEX treatment (Fig 4D). By contrast, the effect of DEX treatment on metastasis was very clear. The abdominal metastasis, involving the spleen, omentum, kidney, GI tract, liver, and diaphragm, was inhibited significantly (Fig 4B) when analyzed by t test by comparing each individual dose with the control or in most of the cases when using ANOVA followed by Dunnett's post-hoc test to compare all doses with the control together. We further confirmed the miR-708 upregulation in the primary ovarian tumors upon DEX treatment (Fig 4E), which accords with our *in vitro* observation that miR-708 expression was upregulated by GC-mediated signaling (Fig 3A). Each single dose of DEX treatment resulted in significantly increased miRNA-708 by Student t test, however, the 50 μg/kg treatment did not further increase the miRNA level and the 3 doses treatment, when compared together, did not show significant proportional increase, despite a significant increase for the 100 μg/kg treatment group compared with the 5 or 50 μg/kg groups. Overall, we demonstrated that GC-mediated signaling has a trend to inhibit ovarian tumor growth, and clearly significantly inhibited abdominal metastasis in the immunocompetent syngeneic mouse model.

### Dexamethasone reduces the expression of various cytokines

**Fig 5:**
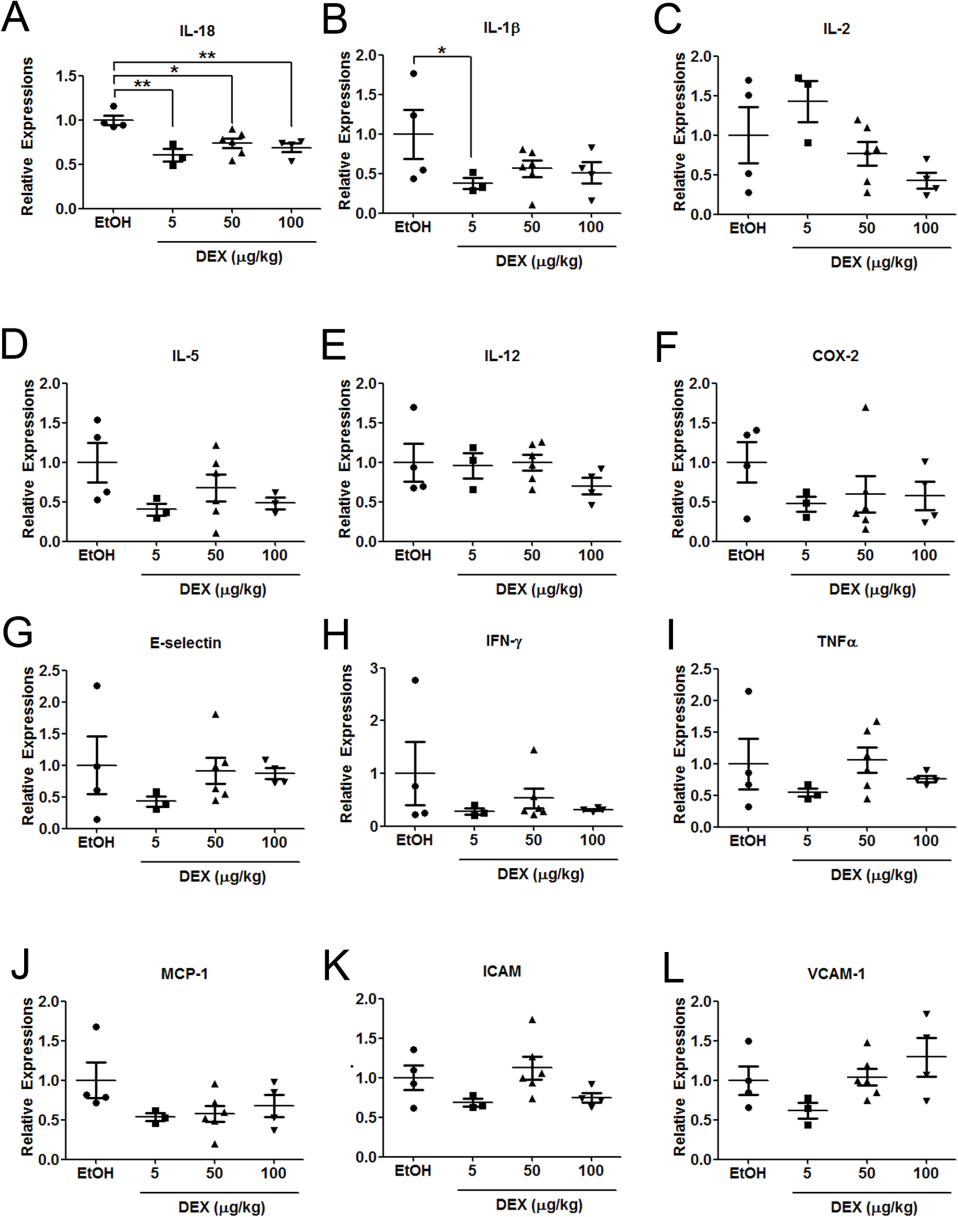
Low-dose DEX inhibits the expression of various proinflammatory cytokines. Expression of IL-18 (A), IL-1β (B), IL-2 (C), IL-5 (D), IL-12 (E), COX-2 (F), E-selectin (G), IFN-γ (H), TNF-α (I), MCP-1 (J), ICAM (K), and VCAM-1 (L) in ovarian tumors engrafted in mice with control or DEX treatment (5, 50, and 100 μg/kg). Data represent as mean ± SEM (n = 3–6 mice per group). ANOVA followed by Dunnett's post-hoc test was used for the statistical test (*P < 0.05; **P < 0.01).

GCs are potent inhibitors of inflammatory processes and are widely prescribed as immune suppression medications [21]. The anti-inflammatory effects of GCs mainly result from the transrepression regulation of the glucocorticoid receptor (GR) through its tethering to proinflammatory transcription factors including activating protein-1 (AP-1) and nuclear factor (NF)-κB [22, 23]. GCs repress a vast number of proinflammatory cytokines such as interleukin (IL)-1β, IL-2, IL-4, IL-5, IL-6, IL-8, IL-12, IL-18, cyclooxygenase (COX)-2, E-selectin, interferon (IFN)-γ, tumor necrosis factor (TNF)-α, monocyte chemoattractant protein (MCP)-1, intracellular cell adhesion molecule (ICAM), and vascular cell adhesion molecule (VCAM) [23]. To investigate whether low-dose DEX suppresses inflammation in ID-8-derived ovarian tumors, we examined the changes in the aforementioned cytokines in in the ovarian tumors engrafted in mice (Fig 5A–5L). DEX downregulated nearly all of the examined cytokines. Among them, the expression of IL-18 was significantly downregulated in all DEX-treated groups, even in the group treated with the lowest dose of DEX (Fig 5A). IL-18, a biomarker of human ovarian carcinoma [24], is known to enhance immunosuppressive responses by impairing the antitumor function of natural killer (NK) cells [25] or promoting the differentiation of monocytes into MDSCs [26], indicating that IL-18 is the key mediator of the interaction between tumor and tumor promoting immune cells in the microenvironment. We also observed that the expression of IL-1β was significantly reduced in the group treated with the lowest dose of DEX, and this reduction was consistently observed in higher dose of DEX treated groups (Fig 5B). Notably, both IL-1β and IL-18 are members of the same structural family (IL-1 family) [27] and their respective precursor forms are inactive until cleaved by caspase-1 activation complexes (inflammasomes) [28]. Neutualization of these two inflammasomes released products, IL-1β and IL-18, has been suggested to be a promising therapeutic agent for tumor suppression in certain cancer types [29], indicating the importance of these two cytokines in the tumor microenvironment. Overall, our data demonstrate that treatment of DEX reduces expression of pro-inflammatory cytokines, especially IL-1β and IL-18, and thus enhances immunosurveillance in the tumor microenvironment and anti-metastatic actions.

### Dexamethasone suppresses the recruitment and maintenance of infiltrating tumor-associated macrophages and myeloid-derived suppressor cells in the tumor sites

**Fig 6:**
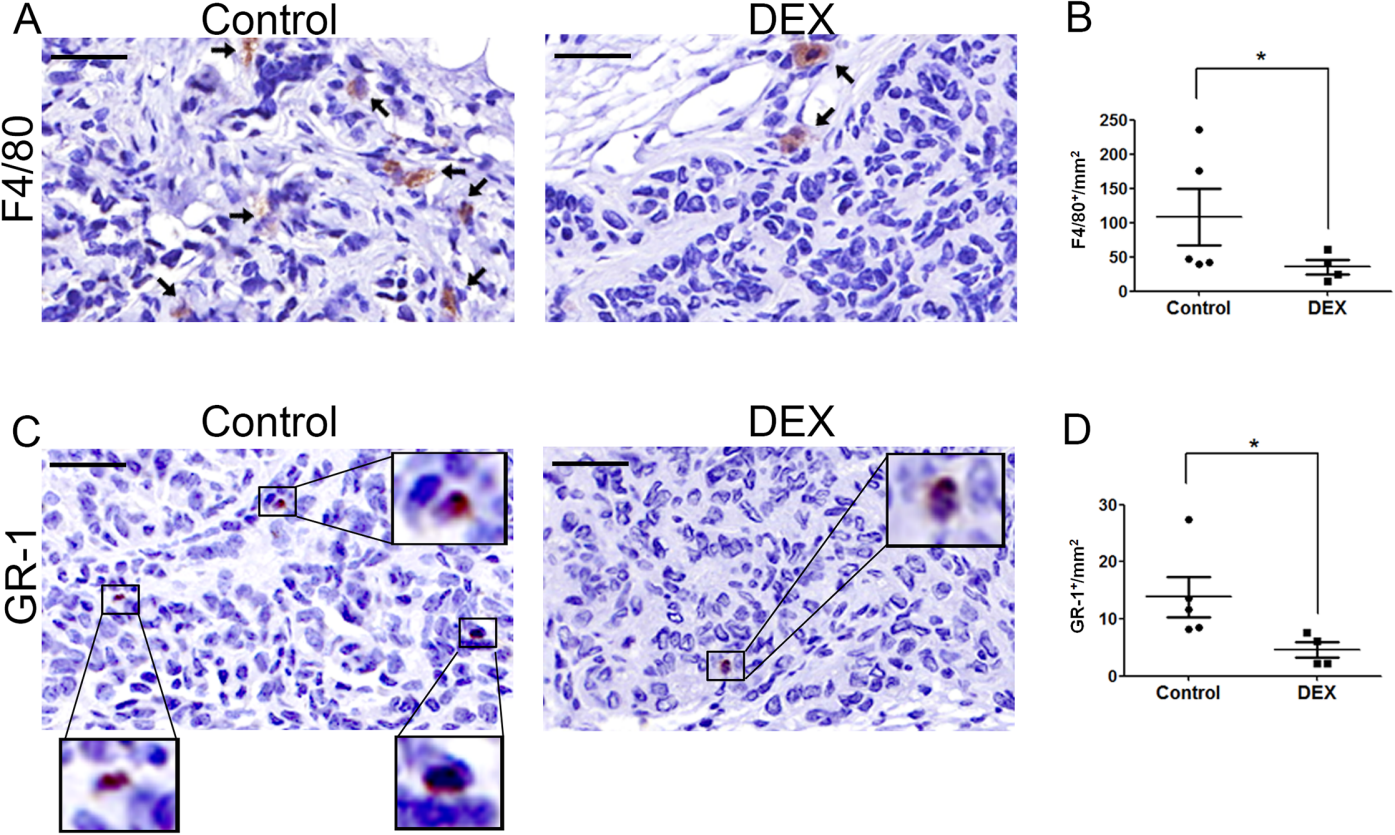
DEX reduces numbers of tumor-associated macrophages and myeloid-derived suppressor cells in the primary ovarian tumor. (A) Representative IHC images stained with the macrophage marker F4/80 and counterstained with hematoxylin in orthotopically implanted ID-8 tumors with or without DEX treatment (5 μg/kg). Arrows indicate F4/80-positive staining of macrophages. Scale bars: 75 μm. (B) The number of F4/80^+^ macrophages per mm^2^ in ID-8 tumors. Data are expressed as the mean ± SEM (n = 4–5 mice per group). Student *t* test was used for the statistical analysis (*P < 0.05). (C) Representative IHC images stained with GR-1^+^ myeloid lineage cells, with hematoxylin counterstaining, in orthotopically implanted ID-8 tumors with or without DEX treatment (5 μg/kg). The GR-1-positive cells were enlarged as indicated. Scale bars: 75 μm (D) The number of GR1^+^ myeloid cells per mm^2^ in ID-8 tumors. Data are expressed as mean ± SEM (n = 4–5 mice per group). Student *t* test was used for the statistical analysis (*P < 0.05).

In ovarian tumors, myeloid cells, including TAMs and MDSCs, are the major determinants of immunosuppression [30, 31]. To investigate the GC-mediated modulation of these immune cell types in the ovarian tumor microenvironment, we determined the numbers of macrophage and myeloid cells in tumors after DEX treatment (5 μg/kg) through immunohistological (IHC) staining by using F4/80 and GR-1 antibodies specific for macrophages and MDSCs, respectively. IHC studies revealed significantly fewer F4/80- and GR1-positive cells in the tumor stroma of the DEX-treated group compared with that of the control group (Fig 6A–6D). Overall, our data suggest that DEX, even at such a low dose, suppressed the expression of proinflammatory cytokines, namely IL-1β and IL-18, and the concordant decrease in MDSCs and TAMs in the tumor stroma. This DEX-mediated modulation of the immune cells in the tumor microenvironment may account, at least in part, for the reduced tumor size and abdominal metastasis.

## Discussion

**Fig 7:**
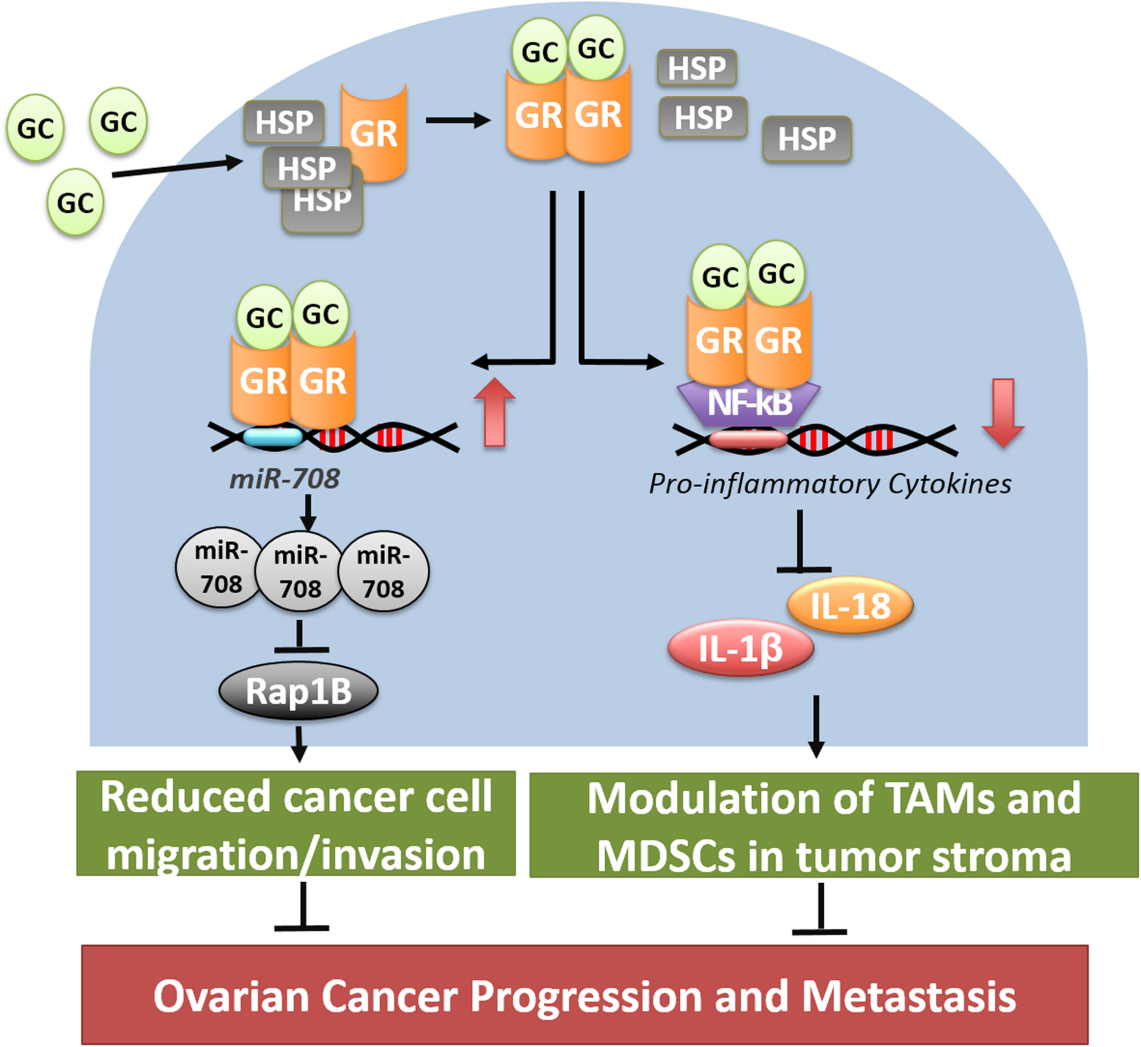
Model of two distinct GCs-mediated signaling pathways suppresses ovarian cancer progression and metastasis. GC-mediated signaling suppresses ovarian cancer progression and metastasis via two distinct pathways. It can induce the expression of miR-708, leading to the depletion of Rap1B and suppression of ovarian cancer metastasis. On the other hand, it can reduce the expression of proinflammatory cytokines, namely IL-1β and IL-18, resulting in the decreased number of MDSCs and TAMs in the tumor stroma and reversion of an immunosuppressive tumor microenvironment.

In this study, we demonstrated that low-dose GCs suppress both ovarian tumor growth and abdominal metastasis in the immunocompetent syngeneic mouse model. In agreement with our previous observation [8], we observed that GC-mediated signaling induced the expression of miR-708, leading to the depletion of Rap1B and suppression of abdominal metastasis in ovarian tumor-bearing mice with an intact immune system. Moreover, GC reduced the secretion of proinflammatory cytokines, namely IL-1β and IL-18. A parallel decrease in MDSCs and TAMs in the tumor stroma manifests the DEX-mediated reversion of an immunosuppressive tumor microenvironment (Fig 7). Overall, our study strengthens the potential therapeutic application of low-dose GCs in ovarian cancer progression and metastasis.

Our previous study showed that GCs suppress ovarian cancer metastasis by inducing miR-708 in the human tumor xenograft in an immunocompromised mouse model [8]. However, recent observations regarding the effects of the tumor microenvironment, particularly immune cells, on tumor progression and metastasis led to the analysis of the effects of GC in ovarian cancer metastasis in immunocompetent mice. Here, we observed that low-dose GCs significantly reduced abdominal metastasis in the syngeneic mouse model (Fig 4B), supporting and extending our previous findings. Moreover, both abdominal metastasis and the size of orthotopically implanted tumors were significantly reduced in the DEX-treated group (Fig 4B). In our previous study, the tumor size in immunocompromised mice in the DEX-treated group was only slightly reduced compared with that in the control group [8]. This differential effect on the growth of primary tumors implies the possible involvement of immune system other than the induction of miR-708 in suppressing tumor growth and metastasis in our current immunocompetent mouse model.

Ovarian tumors comprise both malignant cells and various nonmalignant stromal cells including endothelial cells, fibroblasts, and immune cells. This complex tissue environment, the so-called microenvironment, establishes a bidirectional communication with tumor cells that affects tumor angiogenesis, growth, progression, and metastasis [32, 33]. Studies have indicated that inflammation in the tumor microenvironment is a critical factor for promoting cancer progression [34]. The present study is the first to report that low-dose GCs suppress several proinflammatory cytokines in ovarian tumors (Fig 5), suggesting the therapeutic potential of GCs in modulating the ovarian tumor microenvironment. The GC-mediated suppression of these proinflammatory cytokines may result from the transrepression of NF-κB signaling through the GR [7]. Consistent with these observations, we observed a parallel decrease in MDSCs and TAMs in the tumor sites in the DEX-treated mice (Fig 6). Both MDSCs and TAMs are crucial in immune suppression in the ovarian tumor microenvironment [32]. TAMs facilitate ovarian cancer cell invasion through c-Jun and NF-κB signaling [34], whereas MDSCs inhibit T-cell activation and enhance cancer stem cell gene expression, sphere formation, and metastasis in ovarian cancer [35]. Therefore, the inhibition of MDSCs and TAMs in the tumor sites by low-dose GCs may contribute to the suppression of ID-8 tumor progression and abdominal metastasis.

Studies have reported that cotreatment with GCs in cell culture or a xenograft mouse model inhibits chemotherapy-induced ovarian cancer cell apoptosis [36–38], raising concerns of using GCs as an adjuvant during chemotherapy. Contrastingly, our unpublished data revealed that pre-, co-, or post-treatment of DEX (10–1000 nM) with cisplatin and paclitaxol in a human EOC cell line, SKOV-3, did not inhibit cisplatin and paclitaxol induced-apoptosis. Moreover, clinical studies have reported no significant difference in survival or recurrence between patients with ovarian cancer who received chemotherapy concurrently with GCs and those who did not receive GCs [39, 40]. Additional studies are required to clarify the exact roles of GCs in ovarian cancer progression.

GCs play an integral role in the management of many different physiological conditions [5]. The dosage of GCs used widely varies with the purposes. Patients were administered 50–100 mg of synthetic GCs daily to treat lymphocytic malignancy and 8–20 mg to relieve chemotherapy-induced nausea and vomiting [41]. At these GC dosages, a marked effect on the immune system as well as a multitude of gene regulations can be expected. In this study, we report using low-dose GCs as a therapeutic reagent against ovarian cancer progression and metastasis in a syngeneic mouse model. We revealed that the human equivalent dose of DEX can be as low as 0.4 μg/kg per administration. This dose used for suppressing ovarian cancer progression and metastasis is more than 100-fold less than those currently used in clinical practice, thus minimizing the possible side effects of GC therapy. Future studies are required to elucidate the optimal timing, duration, dosage, and combination therapy as well as the choice of appropriate GCs to develop a customized strategy for treating metastatic ovarian cancer.

## Supporting information

S1 Table Primers and siRNAs used in this study. (DOC)
