# Supplementary material for: Low-dose glucocorticoids suppresses ovarian tumor growth and metastasis in an immunocompetent syngeneic mouse model
Source: PLoS One. 2017 Jun 7;12(6):e0178937. doi: 10.1371/journal.pone.0178937 (PMC5462394; doi:10.1371/journal.pone.0178937)
Supplement: S1 Table — (DOC) [file pone.0178937.s001.doc]

| **qRT-PCR primer sequences (SYBR)** | | | | |
| --- | --- | --- | --- | --- |
|  | | Forward | | Reverse |
| IL1b | | CAACCAACAAGTGATATTC | | ACAGGACAGGTATAGATT |
| IL2 | | CAGGATGGAGAATTACAG | | TACTCTGATATTGCTGATG |
| IL5 | | AAGCAATGAGACGATGAG | | ACGGACAGTTTGATTCTT |
| IL12 | | ACAGATGACATGGTGAAG | | TCTCGTTCTTGTGTAGTTC |
| IL18 | | CGCTTTACTTTATACCTGAA | | TTGTCAACGAAGAGAACT |
| COX2 | | CAGACTCATACTCATAGGAGAG | | GAAGTGGTAACCGCTCAG |
| E-selectin | | CGAGAAGAACGGATAGAGA | | AAACTTTCTGTTCAGTGGAA |
| IFN-γ | | AACTGGCAAAAGGATGGT | | TGTGATTCAATGACGCTTATG |
| TNF-α | | CCAAAGGGATGAGAAGTTC | | GTGGTTTGTGAGTGTGAG |
| MCP-1 | | CTCAGCCAGATGCAGTTA | | TCTCCAGCCTACTCATTG |
| ICAM | | CTCCGTACCTTTGCCATG | | CCTTTCTGAGTCCACTTCTAA |
| VCAM1 | | TACTACACTCACCTTCAT | | TTGGAGAGACTTGGATAA |
| actin | | CGGCATCGTCACCAACTG | | TCTCAAACATGATCTGGGTCATCT |
| **siRNA sequence** | | | | |
| Rap1B | AGACACUGAUGAUGUUCCA | | | |
| **miRNA qRT-PCR primer sequences (Taqman)** | | | | |
| miR-708-RT | | | 5'- GTTGGCTCTGGTGCAGGGTCCGAGGTATTCGCACCA  GAGCCAACCCCAGC - 3' | |
| miR-708 Forward Primer | | | 5'- CGGCGGAAGGAGCTTACAATCTA - 3' | |
| U6-RT | | | 5'-GTTGGCTCTGGTGCAGGGTCCGAGGTATTCGCACCA  GAGCCAACAAAAATAT - 3' | |
| U6 Forward Primer | | | 5'- TTCCTCCGCAAGGATGACACGC - 3' | |
| Universal Reverse Primer | | | 5'- GTGCAGGGTCCGAGGT - 3' | |

**Supplementary Table 1** Primers and siRNAs used in this study
